# Extracellular Vesicles from *Naegleria fowleri* Induce IL-8 Response in THP-1 Macrophage

**DOI:** 10.3390/pathogens11060632

**Published:** 2022-05-30

**Authors:** Sakaorat Lertjuthaporn, Jinjuta Somkird, Kittima Lekmanee, Anyapat Atipimonpat, Kasama Sukapirom, Hathai Sawasdipokin, Supathra Tiewcharoen, Kovit Pattanapanyasat, Ladawan Khowawisetsut

**Affiliations:** 1 Center for Research and Innovation, Faculty of Medical Technology, Mahidol University, Nakhon Pathom 73170, Thailand; sakaorat.ler@mahidol.edu; 2 Graduate Program in Anatomy, Department of Anatomy, Faculty of Medicine Siriraj Hospital, Mahidol University, Bangkok 10700, Thailand; pleng.2486@gmail.com; 3 Center of Excellence for Microparticle and Exosome in Diseases, Research Department, Faculty of Medicine Siriraj Hospital, Mahidol University, Bangkok 10700, Thailand; kittima.lem@gmail.com (K.L.); kasama.suk@mahidol.ac.th (K.S.); kovit.pat@mahidol.ac.th (K.P.); 4 Department of Biochemistry, Faculty of Medical Science, Naresuan University, Phitsanulok 65000, Thailand; anyapata@nu.ac.th; 5 Department of Parasitology, Faculty of Medicine Siriraj Hospital, Mahidol University, Bangkok 10700, Thailand; hathai.noc@mahidol.ac.th (H.S.); supathra.tie@mahidol.ac.th (S.T.)

**Keywords:** extracellular vesicles, *Naegleria fowleri*, exosomes, monocytes, macrophages, pro-inflammatory cytokines

## Abstract

Extracellular vesicles (EVs) released from pathogenic protozoans play crucial roles in host–parasite communication and disease pathogenesis. *Naegleria fowleri* is a free-living protozoan causing primary amoebic meningoencephalitis, a fatal disease in the central nervous system. This study aims to explore the roles of *N. fowleri*-derived EVs (*Nf*-EVs) in host–pathogen interactions using the THP-1 cell line as a model. The *Nf*-EVs were isolated from the *N. fowleri* trophozoite culture supernatant using sequential centrifugation and characterized by nanoparticle tracking analysis and transmission electron microscopy. The functional roles of *Nf*-EVs in the apoptosis and immune response induction of THP-1 monocytes and macrophages were examined by flow cytometry, quantitative PCR, and ELISA. Results showed that *Nf*-EVs displayed vesicles with bilayer membrane structure approximately 130–170 nm in diameter. The *Nf*-EVs can be internalized by macrophages and induce macrophage responses by induction of the expression of costimulatory molecules CD80, CD86, HLA-DR, and CD169 and the production of cytokine IL-8. However, *Nf*-EVs did not affect the apoptosis of macrophages. These findings illustrate the potential role of *Nf*-EVs in mediating the host immune cell activation and disease pathogenesis.

## 1. Introduction

Parasitic diseases, caused by helminths and protozoa remain a worldwide problem threatening human health and well-being. *Naegleria fowleri* infection causes primary amoebic meningoencephalitis (PAM), which is a fatal disease of the central nervous system (CNS) [1,2]. The disease progresses rapidly and death usually occurs within a week. *N. fowleri* is a thermophilic free-living amoeba that is found naturally in freshwater environments such as lakes, rivers, and hot springs or poorly maintained chlorinated swimming pools [3,4]. *N. fowleri* trophozoites enter the human body during swimming or diving in freshwater or inadequately chlorinated pools. The infection by *N. fowleri* occurs when *Naegleria*-containing water enters the nose and subsequently migrates to the CNS along with the olfactory bulbs. *N. fowleri* invades the CNS of the host through a contact-dependent process and secretion of soluble cytolytic factors [5,6,7,8]. Once *N. fowleri* reaches the brain, it elicits a significant immune response by activating the innate immune system. The combinations of the pathogenicity of *N. fowleri* and the intense host immune response are important keys in driving PAM pathology by inducing the destruction of tissue and hemorrhagic necrosis of the brain. 

Host innate immune responses that appear to play a critical role in protecting against *N. fowleri* infection include neutrophils, macrophages, and complements [5,9,10]. Previous studies have described that *N. fowleri* trophozoites activate microglial, a brain-resident macrophage, leading to the production of pro-inflammatory cytokines such as IL-1β, IL-6, TNF-α, reactive oxygen species (ROS), and reactive nitrogen species (RNS) [11]. Microglia may play an important role in host defense against CNS invasion of *N. fowleri*. Although the microglia cells are the main resident macrophage of the brain, the mononuclear phagocyte cells are able to enter the brain, then differentiate into activated macrophages, and may also be involved in some CNS pathological situations. The roles of monocytes and macrophages related to the pathogenesis of *N. fowleri* are still poorly understood.

Extracellular vesicles (EVs) are small membrane vesicles naturally released from the cell which carry biomolecules including proteins, metabolites, DNA, mRNA, micro RNAs, and lipids. EVs are classified into subtypes based on their size, biogenesis, and release pathways. The ectosomes (microparticles, MP/microvesicles, MV) are large EVs derived from plasma membrane while exosomes (Exo) are small EVs originating from endosome [12,13,14]. Based on their cargo, EVs have been proposed to be an important mediator of host–parasite communication. Several studies have revealed that EVs from parasites, such as *Plasmodium* spp. [15,16], *Leishmania* spp. [17], *Trypanosoma cruzi* [18], *Acanthamoeba castellanii* [19,20], *Trichomonas vaginalis* [21], *Giardia duodenalis* [22], *Echinococcus granulosus* [23], and *Fasciola hepatica* [24], cargo parasite-derived biomolecules and deliver them into host cells resulting in modulating the host immune system. A recent study has shown that exosome-like vesicles from *A. castellanii*, a free-living amoeba, can induce the immune response of THP-1 monocytes [20]. Regarding the important roles of EVs, *N. fowleri*-derived EVs may play important roles in driving the disease pathogenesis or activating the host immune responses. As macrophages are one of the important immune cells during PAM pathogenesis, this study aimed to investigate the characteristics of *N. fowleri*-derived EVs (*Nf*-EVs) and their activating effect on monocytes and macrophages. We found that the *Nf*-EVs alter the activation marker expression and induce pro-inflammatory cytokine production by THP-1 macrophages. These data suggested the potential role of *Nf*-EVs in activating the immune response that might be involved in driving the PAM pathogenesis.

## 2. Results

### 2.1. Isolation and Characterization of N. fowleri-Derived EVs 

**Figure 1:**
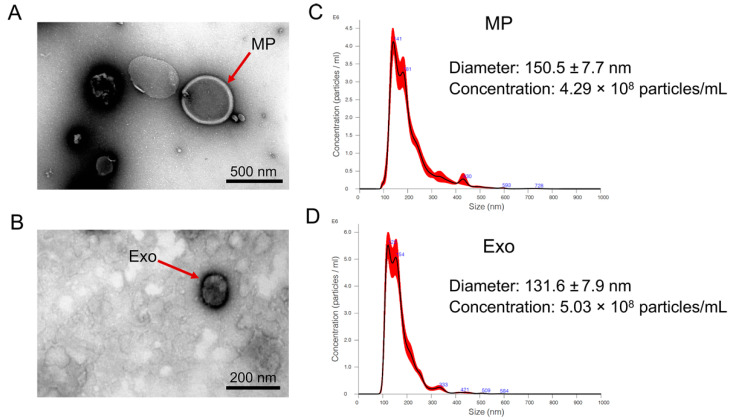
Characterization of *Nf*-EVs. Morphology of *Nf*-EVs using negative staining by transmission electron microscopy (TEM). Cup-shaped vesicles with bilayer membranes are arrowed. (**A**) *Nf*-MP; imaged at the amplification of ×25,000, scale bar represents 500 nm. (**B**) *Nf*-Exo; imaged at the amplification of ×50,000, scale bar represents 200 nm. Nanoparticle tracking analysis (NTA) of *Nf*-EVs. Representative NTA plots showed the mode size distribution and particle concentration of (**C**) *Nf*-MP and (**D**) *Nf*-Exo.

According to the culture protocol, the percentage of viable *N. fowleri* trophozoite culturing in Nelson’s medium containing supplement was 87.4 ± 6.84, while the percentage after culturing in amoeba saline without supplement for 24 h was 75.0 ± 9.41. EVs were isolated from the amoeba saline of *N. fowleri* cultivation by sequential centrifugation. The characteristics of *N. fowleri*-derived microparticles (*Nf*-MP) and *N. fowleri*-derived exosomes (*Nf*-Exo) were confirmed by transmission electron microscopy (TEM) and nanoparticle tracking analysis (NTA). TEM images showed cup-shaped vesicles with a bilayer membrane (Figure 1A,B). The mode size distributions of *Nf*-MP and *Nf*-Exo by NTA analysis were approximately 141.8–172.3 nm and 131.6–151.6 nm, respectively (Figure 1C,D). The average of diameter of *Nf*-MP was approximately 156.8 ± 13.4 nm, while *Nf*-Exo was approximately 141.3 ± 8.3 nm (*n* = 4).

### 2.2. Internalization of Nf-EVs by THP-1 Macrophages

**Figure 2:**
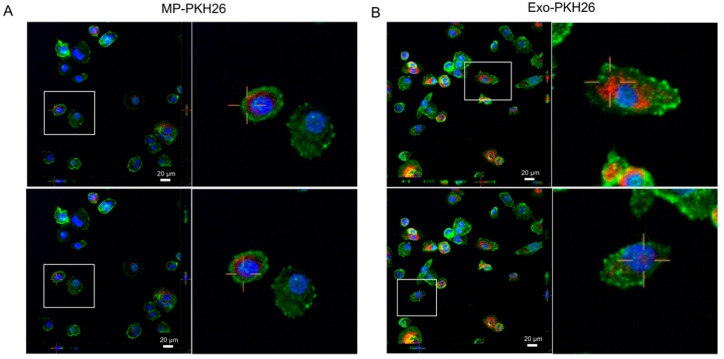
Internalization of *Nf*-EVs by THP-1 macrophages. *Nf*-MP and *Nf*-Exo were labeled with PKH26 red fluorescence marker. Macrophages were incubated with (**A**) PKH26-labeled *Nf*-MP (MP-PKH26; 85 μg/mL), and (**B**) PKH26-labeled *Nf*-Exo (Exo-PKH26; 33 μg/mL) for 2 h, and a confocal analysis was carried out to evaluate *Nf*-EV internalization. Confocal Z-stack projections are shown in all images. The right images are the magnified images of the areas white boxed in the left images. Blue: Nucleus stained with DAPI. Red: PKH26-labeled *Nf*-EVs. Green: F-actin stained with Phalloidin. Scale bar represents 20 μm.

The previous study revealed that THP-1 macrophages and THP-1 derived mature dendritic cells are more efficient at internalizing EVs than THP-1 monocytes or THP-1 derived immature dendritic cells [25]. Therefore, the macrophages were represented as the recipient cells to investigate whether *Nf*-EVs can be uptaken by the target cells. *Nf*-EVs were labeled with PKH26 dye and incubated with macrophages for two hours, followed by observing the internalization using a laser-scanning confocal microscope. The results showed that both *Nf*-MP and *Nf*-Exo were internalized into macrophages when compared to PKH26-PBS control and distributed in the cells (Figure 2 and Supplementary Figure S1).

### 2.3. Nf-EVs Did Not Induce Apoptosis in THP-1 Monocytes and Macrophages 

**Figure 3:**
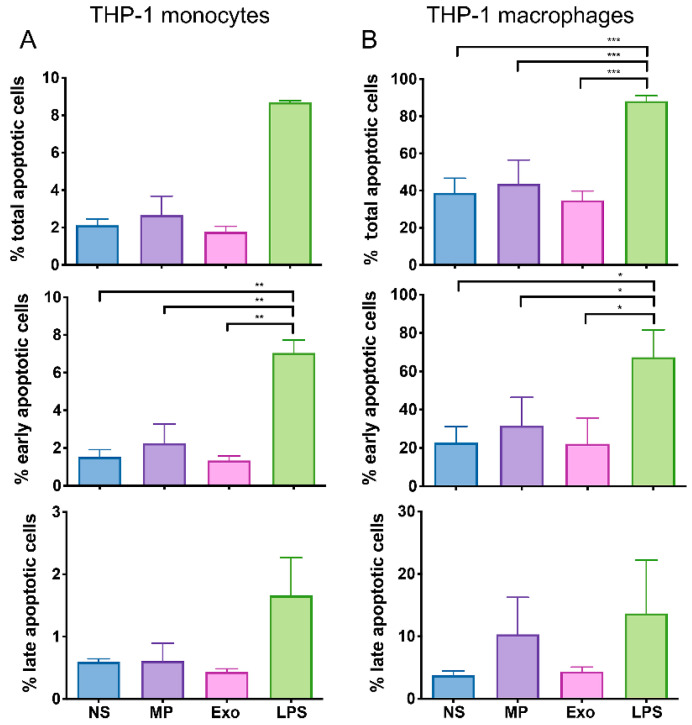
Effect of *Nf*-EVs on apoptosis of THP-1 monocytes and macrophages. THP-1 monocytes and macrophages were treated with 20 μg/mL *Nf*-MP and 20 μg/mL *Nf*-Exo and the expression of annexin V and PI was analyzed by flow cytometry. The percentage of the total, early and late apoptosis of (**A**) THP-1 monocytes and (**B**) THP-1 macrophages were shown. LPS was used as a positive control. Data are mean ± SD from three independent experiments. Statistical analysis was performed by one-way ANOVA with Tukey’s post hoc test. (* *p* < 0.05, ** *p* < 0.01 and *** *p* < 0.001).

Macrophages and microglia cells play an important role in defense against *N. fowleri* infection. However, there is evidence that microglia cells undergo necrosis and apoptosis in response to *N. fowleri* trophozoites infection [26]. To investigate whether *Nf*-EVs triggered the apoptosis of macrophages, we evaluated the cell apoptosis stage by the expression of annexin V and propidium iodide (PI). The annexin V+/PI− cells indicated early apoptotic cells, while annexin V+/PI+ cells indicated late apoptotic cells (Supplementary Figure S2). At 48-h incubation, both *Nf*-MP and *Nf*-Exo did not affect the percentage of total-, early- and late-apoptotic cells of monocytes and macrophages compared to untreated cells (Figure 3). These results indicated that the *Nf*-EVs did not induce monocyte and macrophage apoptosis.

### 2.4. Nf-EVs Alter the Expression Markers on THP-1 Macrophages

Next, to investigate the effect of EVs-derived from *N. fowleri* on the activation of THP-1 monocytes and macrophages responses, the cells were treated with either *Nf*-MP or *Nf*-Exo for 48 h and 72 h, then the expressions of cell surface markers including CD80, CD86, HLA-DR and CD169 (Siglec-1) were determined by polychromatic flow cytometry. All these markers have been found to play an important role in antigen presenting capability to T cells, phagocytosis, cross-presentation and inflammatory conditions on monocytes [27,28,29,30]. The frequencies of cell surface marker-expressing cells and mean fluorescence intensity (MFI) of each cell surface marker were evaluated based on a comparison to their isotype controls (Supplementary Figure S3).

**Figure 4:**
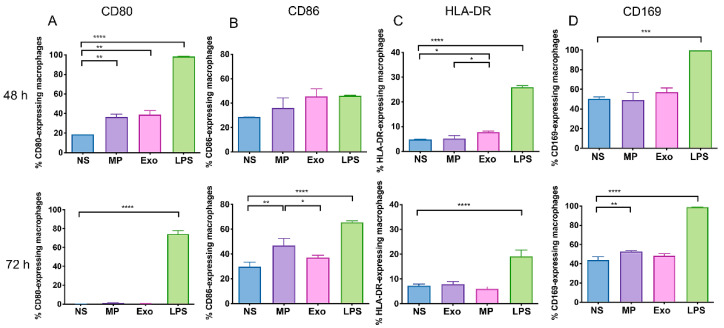
Effect of *Nf*-EVs on the cell surface marker expressions on THP-1 macrophages. THP-1 macrophages were cocultured with 20 μg/mL *Nf*-MP, 20 μg/mL *Nf*-Exo or LPS 25 ng/mL for 48 h (upper panel) and 72 h (lower panel). LPS served as the positive control. The frequencies of expressing cells (**A**) CD80, (**B**) CD86, (**C**) HLA-DR, and (**D**) CD169 were analyzed by flow cytometry. Data are mean ± SD from three independent experiments. Statistical analysis was performed by one-way ANOVA with Tukey’s post hoc test. (* *p* < 0.05, ** *p* < 0.01, *** *p* < 0.001 and **** *p* < 0.0001).

At 48-h exposure time, the baseline expression levels of these protein markers, CD86 and CD169 were highly expressed in non-stimulated macrophages (Figure 4). The incubation with *Nf*-MP and *Nf*-Exo significantly increased the frequencies of CD80-expressing cells compared to the non-stimulated cells (Figure 4A). Moreover, the exposure to *Nf*-Exo at 48 h significantly increased the frequency of HLA-DR-expressing macrophages (Figure 4C). At 72-h exposure time, the CD80 expressions in all cultured groups were downregulated when compared to 48-h incubation. By contrast, the CD86 expressions were still detectable in all cultured groups. The *Nf*-MP-activated macrophages significantly upregulated the expression of CD86 in both frequencies of CD86-expressing macrophages and MFI on macrophages compared to non-stimulated cells (Figure 4B and Supplementary Figure S4). Interestingly, the frequency of CD169-expressed macrophages did not increase after the exposure to *Nf*-EVs at 48 h, but it was significantly increased after *Nf*-MP stimulation for 72 h (Figure 4D). However, there was no statistically significant difference in the frequencies of CD80-, CD86-, HLA-DR-, and CD169- expressing THP-1 monocyte treated with *Nf*-EVs compared to untreated groups (Supplementary Figure S5). 

### 2.5. Nf-EVs Induce the Production of Pro-Inflammatory Cytokines by THP-1 Macrophages

**Figure 5:**
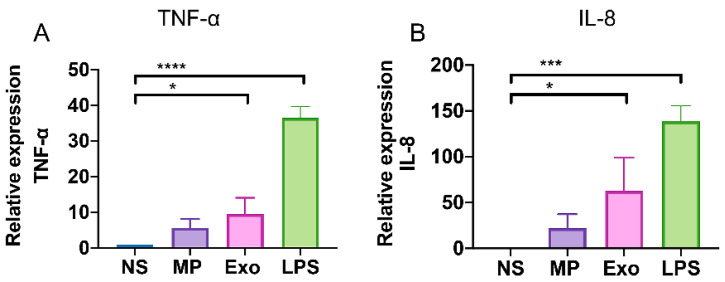
Relative expression of TNF-α and IL-8 in THP-1 macrophages treated with *Nf*-EVs. THP-1 macrophages were cocultured with 20 μg/mL *Nf*-MP, 20 μg/mL *Nf*-Exo or LPS 25 ng/mL for 24 h. The mRNA expression levels of (**A**) TNF-α and (**B**) IL-8 were examined by RT-PCR. Ct values from each condition were compared with GAPDH. The results are represented in mean ± SD obtained from three independent experiments. LPS was used for positive control. Non-stimulated cells (NS) served as the negative control. Statistical analysis was performed by one-way ANOVA with Tukey’s post hoc test. (* *p* < 0.05, *** *p* < 0.001 and **** *p* < 0.0001).

*N. fowleri* trophozoites have been shown to induce pro-inflammatory cytokine production in microglia cells such as TNF-α, IL-1β, and IL-6 [26]. We found that *Nf*-EVs induced the upregulation of CD80 and CD86 which are monocyte-activation markers on THP-1 macrophages. Next, we further explored the effects of *Nf*-EVs on cytokine production by these cells. Firstly, we examined the mRNA expression levels of cytokines including TNF-α, IL-1α, IL-6, IL-10, CXCL10, and IL-8 in macrophages following exposure to *Nf*-MP and *Nf*-Exo for 24 h. The results showed that the expression levels of TNF-α and IL-8 genes in THP-1 macrophages were increased in response to *Nf*-EVs with significantly pronounced in *Nf*-Exo stimulation (Figure 5). These results suggested that the *Nf*-Exo might be a potent factor during *N. fowleri* infection. However, we cannot detect the upregulated levels of IL-1α, IL-6, IL-10, and CXCL10 in the detectable threshold after *Nf*-EV stimulation (data not shown).

**Figure 6:**
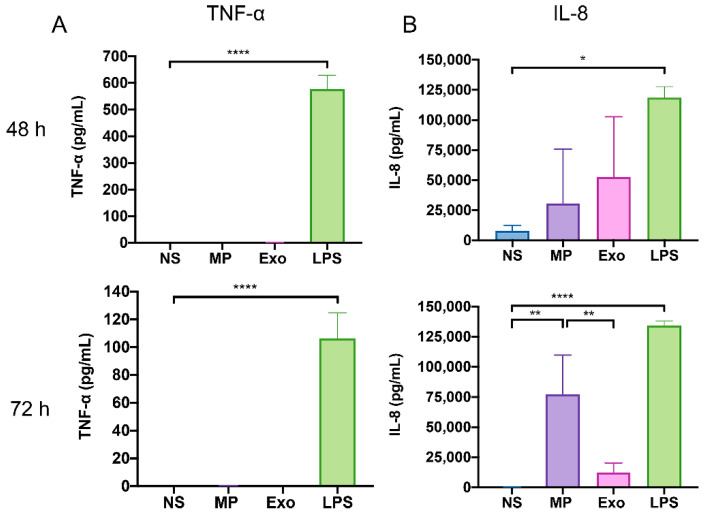
Effect of *Nf*-EVs on the cytokine secretion of THP-1 macrophages. THP-1 macrophages were cocultured with 20 μg/mL *Nf*-MP, 20 μg/mL *Nf*-Exo or LPS 25 ng/mL for 48 h (upper panel) and 72 h (lower panel). Culture supernatants were collected and cytokine levels of (**A**) TNF-α and (**B**) IL-8 were evaluated by commercial ELISA. LPS served as the positive control. Non-stimulated cells (NS) served as the negative control. The results are represented in mean ± SD from three independent experiments. Statistical analysis was performed by one-way ANOVA with Tukey’s post hoc test. (* *p* < 0.05, ** *p* < 0.01 and **** *p* < 0.0001).

To further investigate the cytokine production at the protein level, we measured the TNF-α and IL-8 production of *Nf*-EV activated macrophages at 48-h and 72-h post-activation by ELISA (Figure 6). The IL-8 level at 48-h post-activation was increased to 30,552 ± 45,152 pg/mL and 52,571 ± 50,134 pg/mL (mean ± SD) in macrophages treated with *Nf*-MP and *Nf*-Exo, respectively. Interestingly, the *Nf*-MP activated macrophages upregulated the release of IL-8 to 77,295 ± 32,487 pg/mL (mean ± SD) at 72-h post-activation. It was significantly increased as compared with non-stimulated macrophages (Figure 6B). However, the expression levels of TNF-α from *Nf*-EV activated macrophages were undetectable after 48-h and 72-h activation times (Figure 6A). These results suggested that the *Nf*-EVs, especially *Nf*-MP, might induce macrophage activation resulting in exceeding IL-8 production which is a neutrophil chemotactic factor without inducing the inflammation response. 

## 3. Discussion

Several parasites have shown the ability to go across the blood–brain barrier and infect the human CNS [31]. *N. fowleri* is known to cause severe CNS disease in humans. In *N. fowleri* infection, the pathogenicity of this amoeba is associated with both contact-dependent and contact-independent mechanisms, but the pathogenesis of this infection is still poorly understood. 

In recent decades, the number of studies on EVs released from parasites and parasite-infected cells has dramatically increased, suggesting the significant roles of these EVs in parasite–parasite communication and parasite–host interaction. The EVs from parasites or parasite-infected host cells can induce an efficient immune system [32,33,34,35,36] and modulate the immune response that leads to acceleration of the pathogenesis [37,38,39]. In this study, we demonstrated the characterization of EVs from *N. fowleri* and the effect of these *Nf*-EVs on THP-1 monocytes and macrophages. 

For *Nf*-EV isolation and characterization, we collected the EVs derived from *N. fowleri* trophozoites grown in Page’s buffer saline without supplementation to avoid the protein contamination that might be confounded in *Nf*-EVs. However, the cultural conditions are important. The cultivation in different axenic culture conditions affects growth behavior and morphology, as well as in vitro cytotoxicity and in vivo pathogenicity of amoeba [40]. Thus, the sudden change of the medium might affect the amount and protein composition of *Nf*-EVs. Therefore, it would be better if there were additional processes to decontaminate the proteins from the *Nf*-EVs that are directly isolated from the culture medium supplemented with fetal bovine serum. In addition, the comparative study of EVs derived from different cultivation conditions needs further investigation. According to EV isolation methods, there is still no best method to isolate EVs with a high degree of recovery and specificity for all biofluid types. Differential centrifugation is a commonly used techniques for EV isolation [41,42,43]. We considered that differential centrifugation would be a suitable method to isolate EVs from a large volume of culture supernatant of *N. fowleri* and collect a large amount of *Nf*-EVs. With this isolation method, we showed that *Nf*-EVs present a range from 131 to 172 nm in diameter which was closely similar to the average size of EVs derived from *A. castellanii* [20].

To investigate how *Nf*-EVs interact with monocytes/macrophages and study their role in inducing the immune cell response, the *Nf*-EV internalization data illustrated that macrophages could capture *Nf*-EVs, and the possible mechanism might be active phagocytosis [44]. In a previous report, *N. fowleri* lysate induced apoptosis and necrosis in mouse microglia cells via contact-independent cytotoxicity [45]. The previous study showed that the *N. fowleri* lysate prepared from freeze-thawed parasites contained *N. fowleri* antigen-related protein (nfa-1), the virulence factor located on the pseudopodia of *N. fowleri* [46]. The down-regulation of nfa-1 protein showed a decrease in amoebic cytotoxicity [47]. Moreover, the lysate concentrations used in the cell death study were 0.1, 0.5, or 1 mg/mL. Unlike *N. fowleri* lysate, *Nf*-EVs did not contribute to cell death by the apoptosis pathway in this study. This might be due to the difference in the concentrations and composition of parasite proteins in cell lysate and EVs. Therefore, the study of virulence compositions that *Nf*-EVs carried should be further emphasized.

The classically activated macrophages eliminate pathogens including parasites, bacteria, and viruses through phagocytosis and nitric oxide production. In addition, these activated macrophages express high levels of co-stimulatory molecules and secrete pro-inflammatory cytokines [48,49]. Our results showed that *Nf*-EV stimulation increased the frequencies of CD80-, HLA-DR-, CD86-, and CD169- expressing macrophages. This finding is consistent with a previous study which found that exosomes from *Mycobacterium avium* upregulate the expression of CD80, CD86, HLA-DR, and CD195 on uninfected macrophages [50]. Interestingly, the *Nf*-Exo induced a significant increase of CD80- and HLA-DR- expressing cells after 48 h incubation while *Nf*-MP induced a significant increase of CD80- expressing cells after 48 h and CD86-, and CD169- expressing cells after 72 h. These data seem to indicate that the kinetic responses of macrophages to each *Nf*-EVs subtype were different and *Nf*-MP induced a more prolonged effect on the target cells than *Nf*-Exo. 

In parallel with the cell surface activation, the *Nf*-Exo stimulated a higher level of TNF-α and IL-8 mRNA gene expression at 24 h stimulation and induced a high level of IL-8 protein at 48 h stimulation. By contrast, the *Nf*-MP induced a significant increase in IL-8 level at 72 h stimulation. Although the TNF-α mRNA was upregulated after *Nf*-EV stimulation, the TNF-α protein cannot detect after stimulation. This might be related to the stability of mRNA. The IL-8 mRNA is stable for longer than TNF-α mRNA. In addition, the THP-1 macrophage differentiation by PMA also induced the IL-8 mRNA activation which was stable long-term in the macrophage [51]. Thus, our result showed that IL-8 production but not TNF-α is spontaneously released in non-stimulated cells. Once these macrophages were restimulated with *Nf*-EVs, the IL-8 mRNA can rebound to increase in a shorter time and translate to the high level of IL-8 cytokines. This massive IL-8 production by *Nf*-EV stimulated macrophages might indirectly induce the migration of neutrophils causing accumulation in the brain and involvement in PAM pathogenesis.

In this study, we cannot detect the production of IL-1β, IL-6, and TNF-α in *Nf*-EV stimulated macrophages. Our data were inconsistent with *N. fowleri* trophozoites and the *Nf*-derived products such as Excretory and Secretory Proteins (NfESP) stimulated the expression of proinflammatory cytokines IL-1β, IL-6, and TNF-α from microglia cells [26,45,52]. This might suggest the different *Nf*-derived products that induced the diverse progenitor-derived macrophages and hematopoietic-derived macrophages. Moreover, the studies demonstrated that EVs from *A. castellanii* induce the immune responses of human THP-1 monocytes by secretion of the pro-inflammatory cytokines IL-6 and IL-12 [20] and stimulation with *Toxoplasma gondii* exosomes enhanced the IL-12, TNF-α and IFN-γ production in RAW264.7 cells [34]. Thus, the EVs from each protozoan exhibit unique characteristics and the induced exclusive immune responses are attractive and require further investigation. 

## 4. Materials and Methods

### 4.1. N. fowleri Culture

*N. fowleri* (reference CDC VO3081 strain [53]) was axenically cultivated at 37 °C in Nelson’s medium containing 0.1% (*w*/*v*) liver hydrolysate and 0.1% (*w*/*v*) D-(+)-glucose in Page’s amoeba saline supplemented with 5% fetal bovine serum (FBS) (Gibco, Grand Island, NY, USA), and 1% penicillin/streptomycin (Gibco, Grand Island, NY, USA) in T75-cell culture flask (NEST, Wuxi, China) [40]. At 48–72 h of cultivation and when the amoeba reached about 80–90% confluence, the culture medium was removed. Then, the attached protozoa were washed once with amoeba saline and further cultured in 0.22 µm-filtrated Page’s amoeba saline without adding supplement for 24 h [20]. Then, the supernatant was collected and centrifuged at 1500× *g* for 15 min to remove remaining trophozoites and debris and stored at −20 °C until use for EV isolation.

### 4.2. Cell Culture

The human monocytic leukemia cell line THP-1 (American Type Culture Collection, Manassas, VA, USA) was obtained from Dr. Voravich Luangwedchakarn, Department of Immunology, Faculty of Medicine Siriraj Hospital, Mahidol University, Bangkok, Thailand. THP-1 cells were cultured in complete medium (RPMI 1640 culture medium (Gibco, Grand Island, NY, USA) supplemented with 1% L-glutamine (Gibco, Grand Island, NY, USA), 10% FBS, and 1% penicillin/streptomycin) in 5% CO_2_ at 37 °C. THP-1 monocytes were differentiated into macrophages by incubation with 25 ng/mL of phorbol 12-myristate 13-acetate (PMA) for 48 h followed by culture in complete medium for another 24 h. The incubation of THP-1 monocytes and THP-1 macrophages with 25 ng/mL of bacterial lipopolysaccharides (LPS; Sigma-Aldrich, St. Louis, MO, USA) was used as a positive control for cell activation and cell apoptosis.

### 4.3. Nf-EV Isolation

The *Nf*-EVs were isolated by multi-step differential centrifugation. First, supernatants of *N. fowleri* from Page’s amoeba saline were thawed, filtered through a 1.2 μm Minisart syringe filter (Sartorius, Goettingen, Germany) and centrifuged at 21,000× *g* (Thermo Scientific™ Sorvall RC-6 Plus superspeed centrifuge, Waltham, MA, USA) for 70 min at 4 °C to isolate the *Nf*-MP. The *Nf*-MP pellet was obtained and washed with 0.22 µm-filtrated sterile phosphate buffered saline (PBS) at the same high speed. The remaining supernatant was filtered through a 0.22 μm Minisart syringe filter (Sartorius, Goettingen, Germany) and then ultracentrifuged at 110,000× *g* (Thermo Scientific™ Sorvall WX80 ultracentrifuge, Waltham, MA, USA) for 90 min at 4 °C, the *Nf*-Exo pellet was washed with 0.22 µm-filtrated sterile PBS and finally centrifuged as described in the previous condition. Both *Nf*-MP and *Nf*-Exo were resuspended with 0.22 µm-filtrated sterile PBS and protein concentration was determined by the Bradford method (Bio-Rad Laboratories, Inc., Hercules, CA, USA). An aliquot of isolated *Nf*-EVs was stored at −80 °C until use in the experiments.

### 4.4. Transmission Electron Microscopy

The isolated *Nf*-EVs were visualized by transmission electron microscopy with the protocol as previously described, with some modifications [54]. Briefly, isolated *Nf*-EVs were fixed with 2% glutaraldehyde for 20 min. *Nf*-MP and *Nf*-Exo pellets were deposited on top of the copper grid for 7 min at room temperature. The grid was washed with PBS, followed by distilled water and placed in 2% uranyl acetate for 7 min. Then, the grid was air-dried and imaged by using a Tecnai G2 20 TWIN transmission electron microscope (Field Electron and Ion Company, Hillsboro, OR, USA) at 100 kV.

### 4.5. Nanoparticle Tracking Analysis

The isolated *Nf*-EVs were diluted in 1 mL of 0.22 µm-filtrated sterile PBS and the size distribution and concentration of *Nf*-EVs were analyzed immediately using Nanosight NS300 equipment (Malvern Pananalytical, Worcester, UK) with the following parameters: optimal particle concentration between 20–50 particles per frame, camera level 14, detection threshold level 5. For each sample, five one-min videos were captured. Sample data were analyzed with NTA 3.4 Build 3.4.003 software (Malvern Pananalytical, Worcester, UK).

### 4.6. Nf-EVs Labeling

The isolated *Nf*-EVs were stained with red fluorescent dye PKH26 (Sigma-Aldrich, St. Louis, MO, USA) using the PKH26 red fluorescent cell linker kits for general cell membrane labeling (Sigma-Aldrich, St. Louis, MO, USA) according to the manufacturer’s instructions, with modification. Initially, 6 µL of PKH26 dye was diluted in 1.5 mL of diluent C. Then 30 µL of *Nf*-MP and *Nf*-Exo were diluted in 500 µL of diluent C and gently mixed with dye solution (1:1 *v*/*v*) for 5 min. The labeling reaction was stopped by adding an equal volume of FBS for 1 min. Next, PKH26 labeled EVs were diluted with 10 mL of complete medium and centrifuged at 21,000× *g* for 70 min at 4 °C to pellet the PKH26-labeled MP (MP-PKH26) and centrifuged at. 110,000× *g* for 90 min at 4 °C to pellet the PKH26-labeled exosomes (Exo-PKH26). The PKH26 labeled EVs were further washed with complete medium following the same procedure to remove any free dye, and finally, the MP-PKH26 and Exo-PKH26 were obtained and resuspended in 600 µL of complete medium and used for EVs uptake studies. For the control, labeling was performed as described but without EVs.

### 4.7. Internalization of Nf-EVs with Macrophages

THP-1 monocytes (2 × 10^5^ cells/mL) were seeded on an 8-well chamber slide (Nunc, ThermoFisher™ Scientific, Waltham, MA, USA) and then differentiated to macrophages as mentioned above. The macrophages were cocultured with MP-PKH26 and Exo-PKH26 at 37 °C, 5% CO_2_ for 2 h. The cells were gently washed twice with PBS. The cells were then fixed with 4% paraformaldehyde at room temperature for 30 min and then washed 3 times with PBS. Fixed cells were stained with 1X Green Fluorescent Phalloidin Conjugate working solution using F-actin Staining Kit (Cytopainter, Cat. no. ab112125, Abcam, Cambridge, MA, USA) at 37 °C, 5% CO_2_ for 1 h. Next, the stained cells were washed with PBS. Finally, the stained cells were mounted with DAPI in Fluoromount-G Mounting Medium (Thermo Fisher Scientific, Waltham, MA, USA). The internalization of *Nf*-EVs on macrophages was visualized by a confocal microscope (Nikon ECLIPSE Ti-Clsi4 Laser Unit, City, NY, USA). 

### 4.8. Cell Immunophenotyping and Apoptosis Assay by Flow Cytometry

THP-1 monocytes (0.6 × 10^6^ cells/mL) were seeded on a 24-well cell culture plate and then differentiated into macrophages. THP-1 monocytes and macrophages were cocultured with or without 20 µg/mL of *Nf*-EVs in 5% CO_2_ at 37 °C for 48 h and 72 h. Cells were collected and then washed twice with PBS. Cells were stained with Zombie Aqua™ Fixable Viability Kit (Biolegend, San Diego, CA, USA) to exclude the dead cell population and then incubated with Human TruStain FcX™ solution (Biolegend, San Diego, CA, USA) to block the Fc receptor according to the manufacturer’s instructions. For cell immunophenotyping, cell surface staining was performed by incubating the cells with combinations of the following monoclonal antibodies (mAbs): PE-conjugated anti-human CD80 (Cat. no. 305208, clone 2D10), PE-dazzle594 conjugated anti-human CD86 (Cat. no. 305434, clone IT2.2), PerCP-Cy5.5-conjugated anti-human CD14 (Cat. no. 325622, clone HCD14), APC-conjugated anti-human CD169 (Cat. no. 346008, clone 7-239) and APC-Cy7-conjugated anti-human HLA-DR (Cat. no. 307618, clone L243) antibodies for 15 min at 4 °C in the dark. All mAbs and isotype controls were purchased from Biolegend (San Diego, CA, USA). Cells were finally washed and resuspended in 2% FBS in PBS followed by flow cytometry analysis. For cell apoptosis, cells were stained with APC Annexin V Apoptosis Detection Kit with PI (Biolegend, San Diego, CA, USA) according to the manufacturer’s instructions. Data acquisition and analysis were performed on an LSR II flow cytometer (BD Bioscience, San Jose, CA, USA) using FACSDiva software (BD Bioscience, San Jose, CA, USA). Post-acquisition analysis was performed using FlowJo software version 10 (Becton, Dickinson and Company, Ashland, OR, USA).

### 4.9. Analysis of Cytokine Production by RT-qPCR

Total RNA from THP-1 monocytes and macrophages (1.2 × 10^6^ cells) was isolated by using the RNeasy mini kit (Qiagen, Germantown, MD, USA) according to the manufacturer’s instructions. RNA concentration and purity were determined using a NanoDrop-8000 spectrophotometer (Thermo Fisher Scientific, Waltham, MA, USA). Extracted RNA was used for cDNA synthesis using the SuperScript III First-Strand Synthesis System kit (Invitrogen, Thermo Fischer Scientific, Waltham, MA, USA) on a thermal cycler (T Professional Basic Gradient 96; Biometra, Goettingen, Germany). Real-time PCR was performed with a LightCycler 480 SYBR Green I Master kit (Roche Diagnostics, Mannheim, Germany) according to the manufacturer’s instructions using a LightCycler 480 II Real-Time PCR System (Roche Diagnostics, Mannheim, Germany). The specific primers of human TNF-α, IL-1α, IL-6, IL-8, IL-10, CXCL10 and GAPDH genes are shown in Table 1. PCR condition was performed according to the manufacturer’s instructions. The Ct values were measured in triplicate. Gene expression was normalized to that of GAPDH mRNA in the same samples, using the 2^–∆∆Ct^ method where ΔCt represents the Ct (target gene)—Ct (GAPDH), and ΔΔCt represents the ΔCt (stimulated cells)—ΔCt (non-stimulated cells).

### 4.10. Measurement of Cytokine Concentration by ELISA

The macrophages (0.6 × 10^6^ cells) were cocultured with or without 20 µg/mL of *Nf*-EVs and 25 ng/mL of LPS in 5% CO_2_ at 37 °C for 48 h and 72 h. The culture supernatants were collected for cytokine analysis by enzyme-linked immunosorbent assays (ELISA). The concentrations of TNF-α and IL-8 were determined in duplicate assays using the commercial human TNF-α (Cat. no. 430204; Biolegend, San Diego, CA, USA) and IL-8 ELISA MAX™ Deluxe sets (Cat. no. 431504; Biolegend, San Diego, CA, USA) according to the manufacturer’s instructions. The absorbance was detected at 450 nm using a Synergy H1 Hybrid Multi-Mode Microplate Reader (Biotek Instruments Inc., Winooski, VT, USA). Results were calculated using standard curves generated by a four-parametric logistic (4-PL) curve-fit model.

### 4.11. Statistical Analysis

Data are presented as mean ± SD. Statistical analyses were performed using Prism 8.4.3 software (GraphPad Software Inc., San Diego, CA, USA). One-way ANOVA with Tukey’s post hoc test was used for multiple comparison tests. *p* values < 0.05 were considered statistically significant. 

## 5. Conclusions

In summary, we provided knowledge on isolation and characterization of EVs derived from *N. fowleri*. The work presented here demonstrates that EVs from *N. fowleri* affect macrophages by the upregulation of co-stimulatory molecules expression and IL-8 production. These findings could provide new insights and an important role of *Nf*-EVs in host macrophage response against *N. fowleri* infection. It will be interesting to further study the components of *Nf*-EVs and their potential roles in primary monocytes, macrophages, microglia, and neutrophils which are the major immune cells involved in PAM pathogenesis.

## Figures and Tables

**Table 1:** Primer sequences used for RT-PCR.

Gene	Primer Sequence (5′–3′)		Accession Number
TNF-α	Forward Reverse	CCTGTAGCCCATGTTGTAGCAAA TGAGGAGCACATGGGTGGAG	NM_000594
IL-1α	Forward Reverse	GCGTTTGAGTCAGCAAAGAAGT CATGGAGTGGGCCATAGCTT	NM_000575
IL-6	Forward Reverse	AACCTGAACCTTCCAAAGATGG TCTGGCTTGTTCCTCACTACT	NM_001371096
IL-8	Forward Reverse	ACTGAGAGTGATTGAGAGTGGAC AACCCTCTGCACCCAGTTTTC	NM_001354840
IL-10	Forward Reverse	CCAAGCTGAGAACCAAGACC GGGAAGAAATCGATGACAGC	NM_000572
CXCL10	Forward Reverse	AAGTGGCATTCAAGGAGTACCT GGACAAATTGGCTTGCAGGA	NM_001565
GAPDH	Forward Reverse	ACCCAGAAGACTGTGGATGG TTCAGCTCAGGGATGACCTT	NM_002046

## Data Availability

The data presented in this study are available on request from the corresponding author.

## Supplementary Materials

The following supporting information can be downloaded at: https://www.mdpi.com/article/10.3390/pathogens11060632/s1, Supplementary Figure S1: Representative confocal microscopic images showing the internalization of *Nf*-EVs by THP-1 macrophages. THP-1 macrophages were cocultured with PKH26-labeled *Nf*-MP (MP-PKH26; 85 μg/mL), or PKH26-labeled *Nf*-Exo (Exo-PKH26; 33 μg/mL), or in the absence of EVs (PKH26-PBS control) for 2 h in an 8-wells chamber slide. Blue: Nucleus stained with DAPI, Red: PKH26-labeled *Nf*-EVs, Green: F-actin stained with Phalloidin, Scale bar represents 20 μm. Supplementary Figure S2: Gating strategy of apoptosis on macrophages treated with *Nf*-EVs using Annexin V and PI staining. Supplementary Figure S3: Gating strategy showing CD80, CD86, HLA-DR, and CD169 expression on macrophages treated with *Nf*-EVs. Singlet cells were gated based on FSC-H/FSC-A, SSC-W/SSC-H and FSC-W/FSC-H, then Zombie negative live cells were gated in. Representative histogram plots showing expression markers on macrophages treated with *Nf*-EVs. Light blue histograms correspond to the non- stimulated cells, purple histograms correspond to the *Nf*-MP treated cells, pink histograms correspond to the *Nf*-Exo treated cells, and green histograms correspond to LPS treated cells. Supplementary Figure S4: Effect of *Nf*-MP and *Nf*-Exo on MFI of expression markers of macrophages. Macrophages were cocultured with 20 μg/mL *Nf*-MP or *Nf*-Exo or 25 ng/mL LPS for 48 h (upper panel) and 72 h (lower panel). LPS served as the positive control. The MFI of CD80, CD86, HLA-DR, and CD169 on macrophages were analyzed by flow cytometry. Data are mean ± SD from three independent experiments. Statistical analysis was performed by one-way ANOVA with Tukey’s post hoc test. Supplementary Figure S5: Effect of *Nf*-MP and *Nf*-Exo on expression markers of THP-1 monocytes. THP-1 monocytes were cocultured with 20 μg/mL *Nf*-MP or *Nf*-Exo or LPS 25 ng/mL for 48 h. LPS served as the positive control. The frequencies of CD80, CD86, HLA-DR, and CD169 on THP-1 monocytes were analyzed by flow cytometry. Data are mean ± SD from three independent experiments. Statistical analysis was performed by one-way ANOVA with Tukey’s post hoc test.
