# Supplementary material for: Extracellular Vesicles from Naegleria fowleri Induce IL-8 Response in THP-1 Macrophage
Source: Pathogens. 2022 May 30;11(6):632. doi: 10.3390/pathogens11060632 (PMC9231210; doi:10.3390/pathogens11060632)
Supplement: Supplementary file 1 [file pathogens-11-00632-s001.zip › pathogens-1713694-supplementary.pdf]

Supplementary Materials

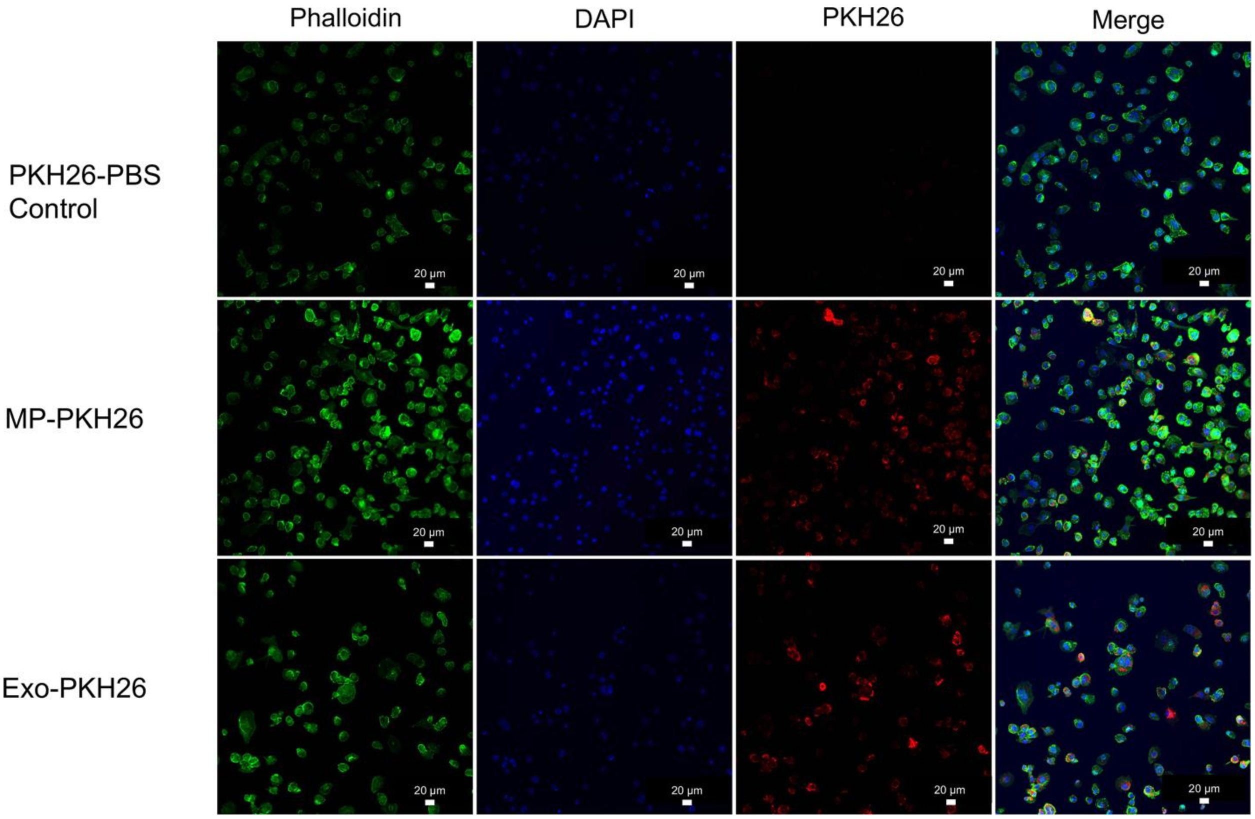

Supplementary Figure S1. Representative confocal microscopic images showing the internalization of *Nf*-EVs by THP-1 macrophages. THP-1 macrophages were cocultured with PKH26-labeled *Nf*-MP (MP-PKH26; 85 μg/mL), or PKH26-labeled *Nf*-Exo (Exo-PKH26; 33 μg/mL), or in the absence of EVs (PKH26-PBS control) for 2 h in an 8-wells chamber slide. Blue: Nucleus stained with DAPI., Red: PKH26-labeled *Nf*-EVs, Green: F-actin stained with Phalloidin, Scale bar represents 20 μm.

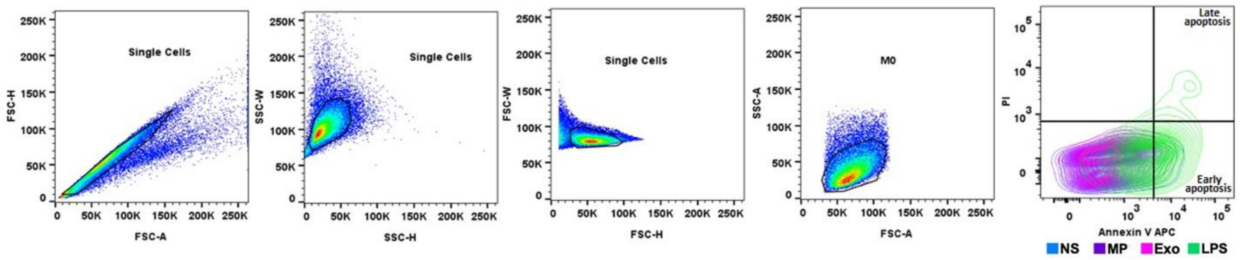

Supplementary Figure S2. Gating strategy of apoptosis on macrophages treated with *Nf*-EVs using Annexin V and PI staining.

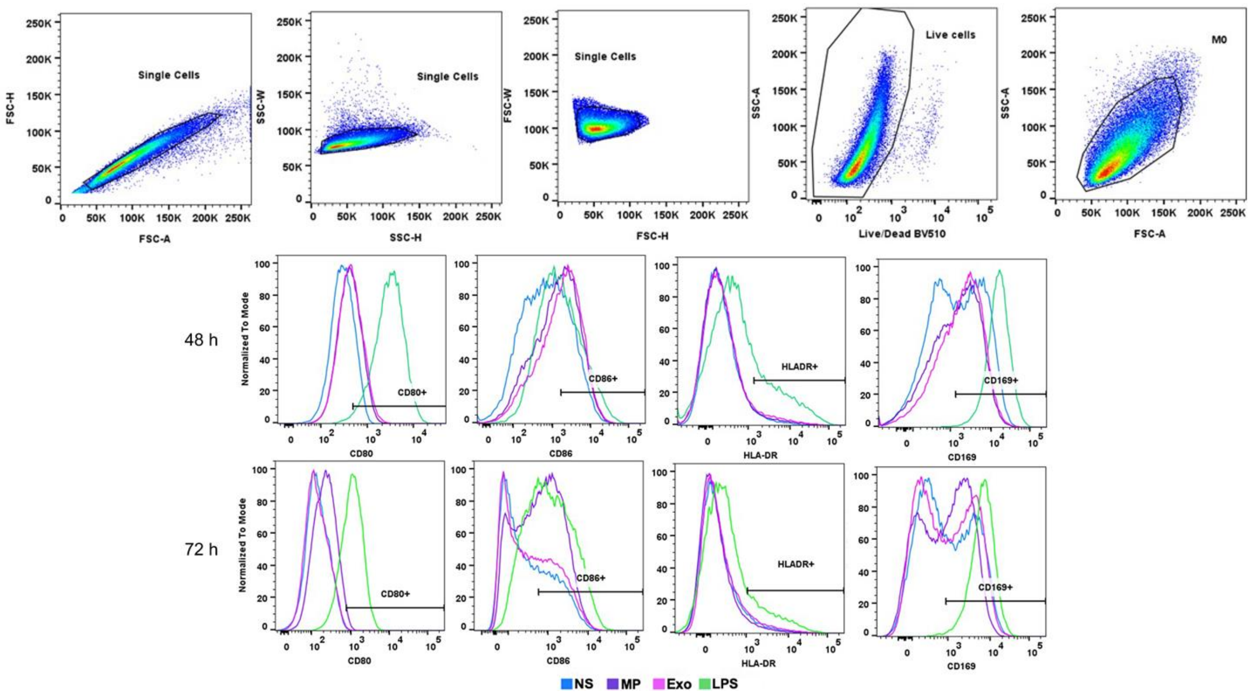

Supplementary Figure S3. Gating strategy showing CD80, CD86, HLA-DR, and CD169 expression on macrophages treated with *Nf*-EVs. Singlet cells were gated based on FSC-H/ FSC-A, SSC-W/SSC-H and FSC-W/FSC-H, then Zombie negative live cells were gated in. Representative histogram plots showing expression markers on macrophages treated with *Nf*-EVs. Light blue histograms correspond to the non-stimulated cells, purple histograms correspond to the *Nf*-MP treated cells, pink histograms correspond to the *Nf*-Exo treated cells, and green histograms correspond to LPS treated cells.

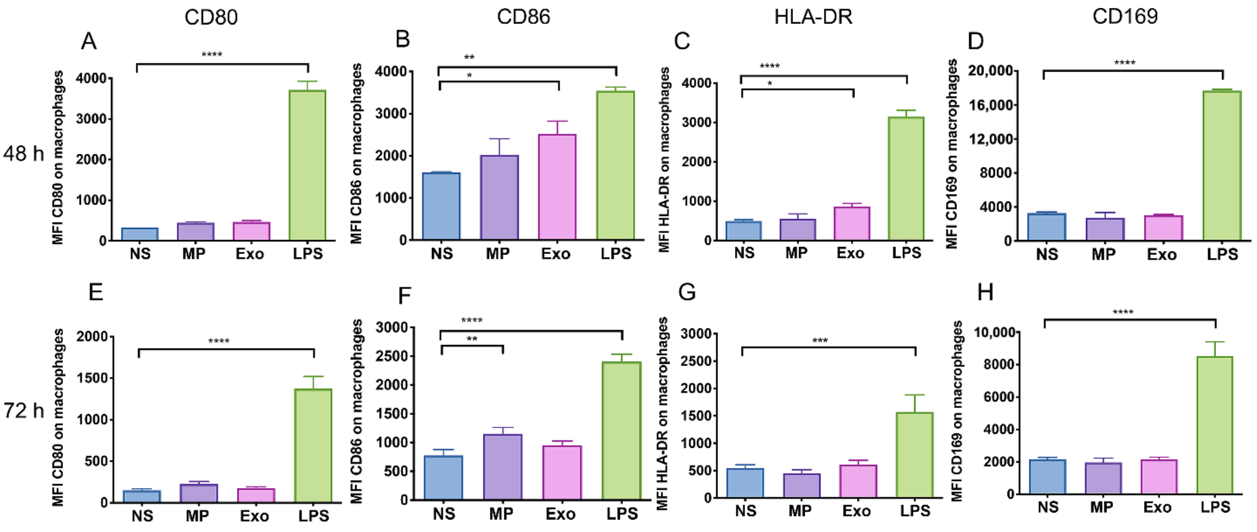

Supplementary Figure S4. Effect of *Nf*-MP and *Nf*-Exo on MFI of expression markers of macrophages. Macrophages were co-cultured with 20 µg/mL *Nf*-MP or *Nf*-Exo or 25 ng/mL LPS for 48 h (upper panel) and 72 h (lower panel). LPS served as the positive control. The MFI of CD80, CD86, HLA-DR, and CD169 on macrophages were analyzed by flow cytometry. Data are mean ± SD from three independent experiments. Statistical analysis was performed by one-way ANOVA with Tukey's post hoc test. (* $p < 0.05$, ** $p < 0.01$, *** $p < 0.001$ and **** $p < 0.0001$)

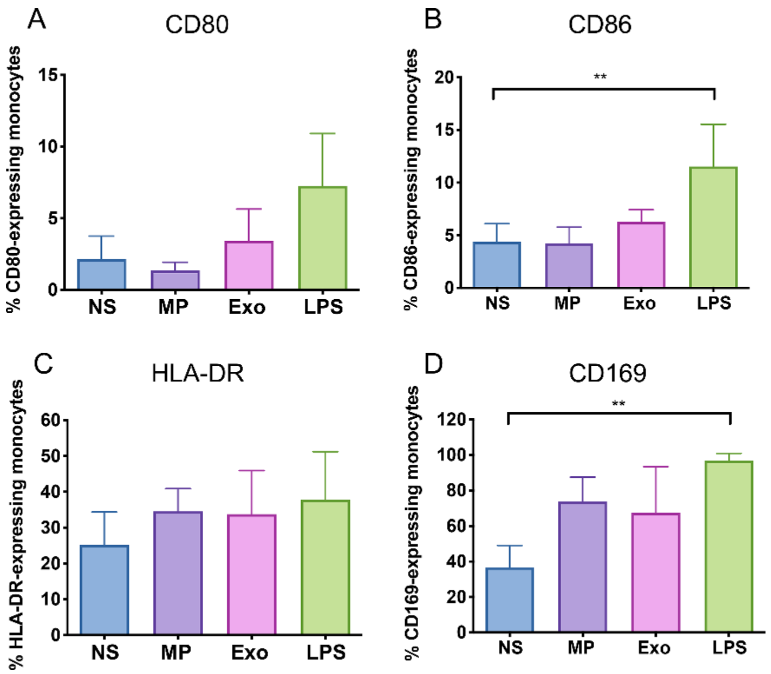

Supplementary Figure S5. Effect of *Nf*-MP and *Nf*-Exo on expression markers of THP-1 monocytes. THP-1 monocytes were cocultured with 20 µg/mL *Nf*-MP or *Nf*-Exo or LPS 25 ng/mL for 48 h. LPS served as the positive control. The frequencies of CD80, CD86, HLA-DR, and CD169 on THP-1 monocytes were analyzed by flow cytometry. Data are mean ± SD from three independent experiments. Statistical analysis was performed by one-way ANOVA with Tukey's post hoc test. (** $p < 0.01$)
